# Enhanced Muscle Flavor in Male Chinese Mitten Crab (*Eriocheir sinensis*) Driven by Feed-Induced Reconfiguration of Intestinal Volatile Compounds

**DOI:** 10.3390/ani15213101

**Published:** 2025-10-25

**Authors:** Jin Cen, Bo Liu, Qunlan Zhou, Xiaochuan Zheng, Gangchun Xu, Hongyan Tian, Linghong Miao, Huiming Ding, Yongfeng Zhao, Cunxin Sun

**Affiliations:** 1 Wuxi Fisheries College, Nanjing Agricultural University, Wuxi 214081, China; 2 Key Laboratory of Freshwater Fisheries and Germplasm Resources Utilization, Ministry of Agriculture and Rural Affairs, Freshwater Fisheries Research Center, Chinese Academy of Fishery Sciences, Wuxi 214081, China; 3 School of Marine and Bioengineering, Yancheng Institute of Technology, Yancheng 224000, China; 4 Suzhou Yangcheng Lake Modern Agricultural Development Co., Ltd., Suzhou 215100, China

**Keywords:** *Eriocheir sinensis*, E-nose, GC–IMS, flavor, intestinal volatiles

## Abstract

**Simple Summary:**

The aquaculture industry faces a critical challenge: replacing unsustainable iced trash fish (IF) with formulated feed (FF) without compromising the prized flavor of the Chinese mitten crab (*Eriocheir sinensis*). While the muscle flavor is paramount, its formation mechanism, particularly the potential transfer and deposition of volatile compounds from the intestinal chyme—a crucial flavor reservoir influenced by diet—remains poorly understood. This study investigated the dual impact of FF replacement on both the intestinal and muscle volatile profiles. Using advanced flavoromics (E-nose and GC–IMS), we discovered that FF significantly altered the intestinal volatile landscape, which in turn promoted the deposition of key sweet and aromatic compounds in the muscle, ultimately enhancing the overall sensory perception. Our findings reveal the gut’s role as a flavor modulation hub and demonstrate that formulated feed is not merely a sustainable alternative to finite marine resources but can actively steer flavor quality improvement. This provides a strategic, dual-benefit approach for crab aquaculture.

**Abstract:**

The traditional use of iced trash fish (IF) in Chinese mitten crab (*Eriocheir sinensis*) aquaculture raises sustainability concerns, but the shift to formulated feeds (FF—a commercial compound feed specifically designed to meet nutritional requirements by blending multiple ingredients and containing a balance of nutrients) is often hindered by fears of compromising its prized flavor. This study aimed to comprehensively evaluate whether a commercial formulated feed could effectively replace IF without diminishing flavor quality, hypothesizing that FF would alter the intestinal volatile profile, thereby influencing muscle flavor. Male crabs were fed either IF or FF for eight weeks. Muscle flavor was assessed using sensory evaluation, electronic nose (E-nose), and gas chromatography–ion mobility spectrometry (GC–IMS). Volatile compounds in intestinal chyme were also analyzed by GC–IMS to explore potential transfer mechanisms. The results indicated that crabs fed with FF showed higher sensory scores for sweetness. Additionally, the E-nose analysis revealed a clear separation trend between dietary groups and showed markedly higher sensor response values for aromatic compounds, biogenically derived compounds and Maillard reaction products, sulfur-containing organic compounds, aliphatic hydrocarbons, total volatile organic compounds, alcohols and organic solvents, and alkenes in the FF group compared to the IF group. Thirty-four volatiles were discovered in the muscle. Statistical analysis (independent samples t-test) showed that the FF group exhibited significantly elevated levels of 3-methylbutanal-M, propanal, (E)-2-pentenal, 2,3-pentanedione, and pentan-1-ol-M, whereas the IF group exhibited significantly elevated levels of 2-hexanone, dihydro-2(3H)-furanone, butyl acetate, ethyl 2-methylpropanoate, and phenol (*p* < 0.05). Fourty-eight volatiles were identified in the intestinal chyme. Propanal and ethyl 2-methylpropanoate were the dominant odor contributors based on correlation network analysis. Strong correlations were identified between the flavor profiles of intestinal chyme and muscle, suggesting a potential transfer or transformation of volatiles. This work provides a scientific basis for optimizing aquafeed formulations to ensure sustainable crab production without sacrificing end-flavor quality.

## 1. Introduction

Chinese mitten crab (*Eriocheir sinensis*) is a popular aquatic food among most Chinese customers because of its distinct perfume and intense umami flavor. *E. sinensis* cultivation has become a potential freshwater fishery and one of the greatest commercial crustacean businesses, resulting in the fast development of crab farming, particularly along China’s east coast. The yearly output of *E. sinensis* has increased substantially from 775,887 tonnes in 2020 to 888,629 tonnes in 2024 [1]. The fattening phase of *E*. *sinensis*, also known as the “final growth stage” or “pre-harvest maturation period,” is a critical period in aquaculture aimed at enhancing body weight, hepatopancreas nutrient accumulation, and gonadal development to meet market demands for quality and flavor. This phase typically occurs after the crab undergoes its final molting (post-reproductive molting) and lasts for 4–6 weeks prior to harvest. In the earthen pond culture model, *E. sinensis* was fed with iced trash fish and natural feed sources such as soybean and corn in the fattening phase [2]. However, trash fish contaminates the water of aquaculture systems owing to its perishable and relatively poor utilization, thereby severely compromising the healthy and sustainable development of *E. sinensis* cultivation [3]. As a result, substituting iced trash fish with formulated feed has become an unavoidable trend in the culture of *E. sinensis*. Farmers in China are also interested in the utilization of adequate formulated feed in Chinese mitten crab farming.

A previous study has proved that formulated feed replacing iced trash fish has no significant effect on the survival rate, growth performance, and reproduction of *E. sinensis* [4]. Based on China’s 2024 Chinese mitten crab production of approximately 894,400 metric tons, with a feed conversion ratio (FCR) of about 2.5 when using formulated feed throughout the farming cycle, the annual production level of formulated feed required to meet the demands of China’s crab industry would be approximately 2.236 million metric tons. Nevertheless, systematic studies have shown significant differences in the flavor quality between crabs fed iced trash fish and those fed formulated feed. *E. sinensis* reared with iced trash fish have higher ω-3/ω-6 polyunsaturated fatty acid (PUFA) ratio and essential fatty acid levels than those reared with formulated feed, but show no significant effects on free amino acid content [2,5,6]. Additional research also found that diet types significantly altered volatile and non-volatile active components in female *E. sinensis*. Crabs fed with formulated feed had higher sensory evaluation scores than those fed with trash fish and freshwater snails [7]. Crab taste is derived from hundreds of molecules, including aldehydes, ketones, alkanes, alcohols, aromatics, furans, sulfur-containing compounds, nitrogen-containing compounds, and other compounds, which are formed from flavor precursors [8,9]. It is well-established that volatile compounds, including trimethylamine, (Z)-4-heptenal, 3-methylbutanal, heptanal, 2-nonanone, and (E)-2-nonenal, play a pivotal role in shaping the distinctive crab aroma profile that is ultimately perceived and accepted by consumers [10]. Fatty acids and amino acids in feed are important sources of flavor precursors. Studies have shown that volatiles or flavor precursors, such as terpenes and diterpenoids, can be transferred to the muscle from the feed [9]. Still, the transformation mechanism of volatile substances from feed to meat remains unclear. Limited research has pointed out that volatile compounds in feed could be transferred into tissue by the action of intestinal microorganisms [11,12]. However, the correlation between the volatiles in the diet after the action of the gut microbes and the volatiles in the muscles of *E. sinensis* remains to be explored.

The advancement of aquatic product taste research should be attributed mostly to the progress of research methodologies, such as the isolation, detection, and characterization of odor molecules from volatile mixtures. The electronic nose (E-nose) is a device made up of sensors that can respond to diverse combinations of total volatile organic compounds in complicated food samples [13]. The E-nose demonstrates significant advantages in meat flavor detection by providing high sensitivity and objectivity through sensor arrays that capture and differentiate complex total volatile organic compounds (VOCs), such as aldehydes, alcohols, and sulfides, which are critical for aroma profiling. It effectively discriminates flavor attributes across different processing conditions, muscle types, or geographical origins using chemometric models like PCA and PLS, overcoming the subjectivity and time constraints of traditional sensory evaluations. There have been successful cases of E-nose use in the flavor identification of crab muscles, hepatopancreas, and gonads [14,15]. Hence, the E-nose is adequate to distinguish flavor profiles of crab fed with formulated feed and iced trash fish without subjective factors and judgments. Because of its quick reaction, high sensitivity, ease of operation, and cheap cost, gas chromatography–ion mobility spectrometry (GC–IMS) is a strong technology for the separation and sensitive detection of volatile organic molecules [16]. GC–IMS technology has also been successfully used in aquaculture nutrition. Xu et al. applied GC–IMS to identify the change in muscle flesh of *Procambarus clarkii* fed with different protein sources [17]; Cui et al. applied GC–IMS to investigate dietary phospholipids on volatile flavor compounds in *Haliotis discus hannai* [18]; Chi et al. applied GC–IMS to characterize the filet quality of *Megalobrama amblycephala* fed with ferulic acid and oxidized oil [19]. However, the application of GC–IMS on the characterization of *E. sinensis* flavor has been rarely reported.

Given this, the authors hypothesized that the flavor of *E. sinensis* would be affected by formulated feed and iced trash fish. Various volatiles such as aldehydes, ketones, alcohols, and esters may be converted from volatiles or precursors in the intestine. Thus, the present study aimed to evaluate the changes in odor using E-nose and GC–IMS when crabs were fed with formulated feed instead of iced trash fish for fattening. Further, the correlation of volatiles between the intestine and muscle was measured to evaluate the action of intestinal microbe in the muscle flavor. The data may help the application of formulated feed in the whole culture period of *E. sinensis*.

## 2. Materials and Methods

### 2.1. Animal Ethics

The care and management of animals adhered to the criteria established by the Animal Research Institute Committee of Freshwater Fisheries Research Center. This work from the Animal Research Institute of Freshwater Fisheries Research Center has been accepted by the Committee (permit number: FFRC 2022-007).

### 2.2. Experimental Crabs and Culture Conditions

The experiment was conducted at Suzhou Yangcheng Lake Modern Agriculture Development Co. Ltd. (Suzhou, China). In early October, 180 pond-reared male crabs were cultured in earth ponds outside and fed a formulated diet for acclimation. After one week of domestication, crabs of similar size (average 112.65 ± 1.57 g) were randomly distributed into six equal-sized earth ponds (6.0 m × 6.0 m × 1.5 m), with 30 crabs per pond. All crabs used in the experiment had already undergone the reproductive molt. Consequently, they did not undergo any further molting during the entire subsequent 8-week fattening trial. The commercial formulated feed (FF) and iced trash fish (IF) were randomly assigned to crabs in triplicate ponds. Plastic boards surrounded each pond to prevent escape. The commercial formulated feed was prepared as follows: the major raw materials were ground to pass through an 80-mesh sieve, weighed, and mixed step by step. Lipids and 30% water were then added and thoroughly blended. The mixture was pelleted (2.0 mm pellet diameter) using a twin-screw extruder (Model F-26, Guangzhou HGKJ Light-Electro-Mechanical Technology Co., Ltd., Guangdong, China). The pellets were subsequently cooked in a steam cabinet at 85 °C for 15 min for gelatinization and pasteurization. Finally, the feed was air-dried in a cool, shaded area and stored at −20 °C until use. The formulation of FF is shown in Table 1. Crabs were fed approximately 3% of their body weight twice daily (8:00, and 16:00) for eight weeks. The iced trash fish (IF), used as the control diet in this experiment, was primarily composed of anchovy (*Engraulis japonicus*). Its proximate composition (on a wet weight basis) was as follows: moisture 75%, crude protein 16%, crude lipid 5%, and ash 3%. This ratio was a little bigger than the amount of diet consumed by crabs in 1 h. Throughout the experiment, a water depth of 1 m was maintained, with 30% of the water in each pond exchanged every three days. Water temperature ranged from 28 to 30 °C, pH fluctuated between 7.2 and 7.4, dissolved oxygen was maintained above 5.0 mg/L, and total ammonia nitrogen and nitrite was kept <0.2 and 0.005 mg/L, respectively.

After the 8-week fattening period, crabs were anesthetized on ice for 10 min. Fifteen crabs from each pond (n = 3 ponds per diet) were sampled and cooked using live steam at 100 °C for 20 min in a steamer. After cooking, the crabs were used for consumer sensory evaluation. Another three crabs’ muscles from each pond were collected for E-nose and GC–IMS analysis. Each crab was then dissected dorsally using sterile surgical tools. The entire intestinal tract was carefully excised and placed in a sterile Petri dish. The intestinal chyme was collected by gently squeezing the tract with sterile forceps or by flushing it with a minimal volume of sterile physiological saline (0.85% NaCl) into a sterile microcentrifuge tube. For biological replication, chyme from three crabs within the same experimental pond was pooled into one composite sample. Samples were immediately snap-frozen in liquid nitrogen and subsequently stored at −80 °C until further analysis for volatile compounds via GC–IMS.

### 2.3. Consumer Sensory Evaluation

According to Ding et al. [20], the crabs were steamed at 100 °C for 20 min using a steamer. The sensory evaluation was conducted using a consumer panel consisting of 30 untrained students recruited from Nanjing Agricultural University (aged 18–30 years, with no allergies to Chinese mitten crab). Although untrained, all participants were briefed on the meaning of each attribute and the use of the scale prior to evaluation. The muscle samples from each replicate pond (n = 3 ponds per diet) were used. Each muscle sample from the three replicate ponds per diet was assigned a unique three-digit random code. The panelists were not informed about the sample identities (FF or IF group) or the experimental hypotheses. The coded samples were presented to each panelist in a fully randomized order. This randomization was unique for each panelist to effectively minimize any potential serving-order bias or carry-over effects. Samples were served in opaque polyethylene tasting cups at a warm temperature (approximately 40 °C) to ensure optimal aroma release. A standard portion size of approximately 5 g was provided for each evaluation. The intensity of salt taste, sweet taste, umami taste, fishy odor, meaty odor, fatty odor, and grassy odor was rated using a 5-point intensity scale, where 1 = very weak, 2 = weak, 3 = moderate, 4 = strong, and 5 = very strong.

### 2.4. Electronic Nose Analysis

Each treatment group comprised three ponds, with three crabs per pond, resulting in a total of nine biological replicates for E-nose analysis. The iNose E-nose system (FOX4000, Alpha M.O.S, Toulouse, France) was used to determine the flavor volatiles. According to Zhang et al. (2016), the method was slightly modified [7]. Thirteen metal oxide semiconductors sensors were equipped in the E-nose system. The details of the sensor array are as follows: S1, aromatic compounds; S2, sulfides; S3, hydrogen and combustible gases; S4, broad-spectrum polar volatiles: organic acid esters, terpenes, and microbial fermentation products; S5, biogenically derived compounds and Maillard reaction products; S6, sulfur-containing organic compounds; S7, oxygenated derivatives of aliphatic hydrocarbons; S8, nitrogen oxides, ammonia, and low-molecular-weight amines; S9, aliphatic hydrocarbons; S10, total volatile organic compounds; S11, alcohols and organic solvents; S12, alkenes; S13, broad-spectrum volatiles from thermal food processing. For the cooked sample, 2.0 g of tissue was placed in a 10 mL precision thread vial for 10 min of headspace equilibration at 40 °C. After injecting 250 L of the sample’s headspace gas for 1 s, the sensor signals were collected 120 s later. To maintain the samples’ freshness, each sample was put on a sample plate at 4 °C. Each sample was measured three times independently.

### 2.5. Volatile Compound Analysis by GC–IMS

Muscle and intestinal chyme samples from all three crabs within the same pond were pooled together to form a single composite sample per pond for GC–IMS analysis. For GC–IMS analysis, the sampling mass was 2 g for both treatment groups. Each blended muscle sample was tested twice.

FlavourSpec from G.A.S. (Gesellschaft für Analytische Sensorsysteme mbH, Dortmund, Germany) was used for analysis. These studies relied on gas chromatography (Agilent Technologies, Palo Alto, CA, USA) connected to a drift time IMS cell with a CombiPal GC autosampler (CTC Analytics AG, Zwingen, Switzerland). Minor adjustments were made to the GC–IMS study published previously [16]. The samples were put in a 20 mL headspace glass sampling vial and heated at 100 °C for 15 min. Following this, a 500 L volume of headspace was automatically injected into the heated injector using a 110 °C syringe in splitless injection mode. For chromatographic separation at 60 °C, a GC fitted with an FS-SE-54-CB-1 (15 m × 0.53 mm, 1 m) column was used. Nitrogen with a purity of 99.99% was employed as the carrier gas, with the following programmed flow: beginning flow of 2 mL/min, maintained for 2 min, ramped up to 100 mL/min in 18 min, and sustained for 10 min.

IMS conditions were as follows: tritium ion source (6.5 keV), positive ion mode, drift tube length of 9.8 cm, tube linear voltage of 400 V/cm, and drift gas flow rate of 150 mL/min (nitrogen, purity 99.999%). The drift tube temperature was 45 °C. Using n-ketones C4-C9 as external standards, retention indices (RI) were computed for each compound.

### 2.6. Statistical Analysis

The volatile components were gathered and processed using the program LAV-Gallery Plot 2.2.0 (G.A.S, Dortmund, Germany). During the first phase, the retention time, drift time, and ion signal intensity emerged, and prospective markers were identified by comparing response differences. As the dietary treatment was applied at the pond level, the pond was considered the experimental unit for all statistical analyses (n = 3 ponds per dietary treatment). The variance of these pond-level mean values across dietary groups was then examined using an independent-sample *t*-test in SPSS 20.0, with *p* < 0.05 denoting significant differences. All data were expressed as the mean ± the standard error (SE) of the pond replicates (n = 3). In the subsequent stage, chemometric techniques were applied to the pond-averaged data sets to uncover hidden information inside massive data sets. Principal component analysis (PCA), advanced heatmap plots, clustering correlation heatmap with signs, and correlation network were generated by the OmicStudio tools available at https://www.omicstudio.cn (accessed on 20 November 2024).

## 3. Results

### 3.1. Consumer Sensory Evaluation

**Figure 1:**
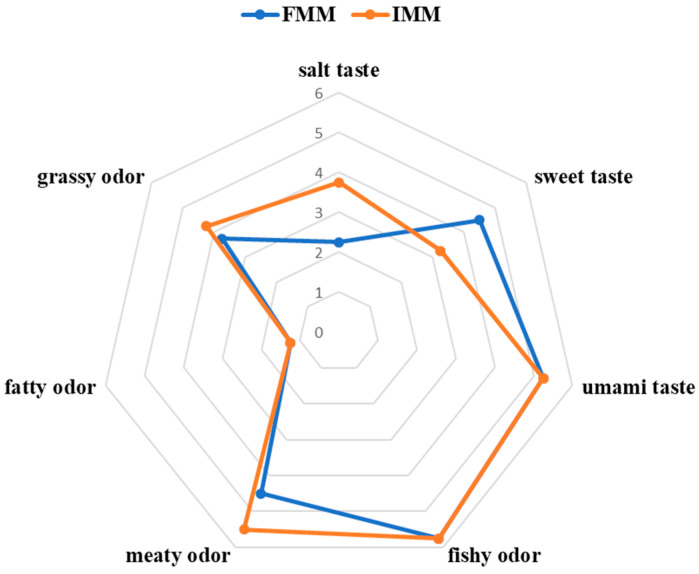
Consumer preference for the muscle of the Chinese mitten crab.

Figure 1 showed the sensory intensities of salt taste, sweet taste, umami taste, fishy odor, meaty odor, fatty odor, and grassy odor of the muscle of Chinese mitten crab. The FMM group had higher scores for sweet taste, while the IMM group had higher scores for salt taste and meaty taste.

### 3.2. E-Nose Analysis of the Crab Fed with Different Diets

**Figure 2:**
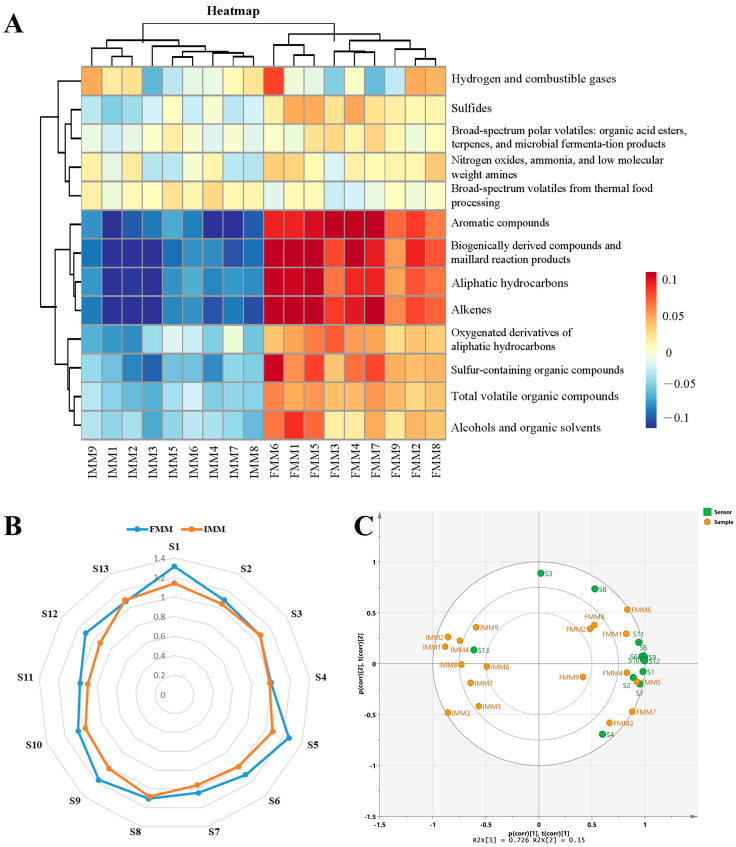
Odor profiles of crab muscle samples by electronic nose. (**A**): the heatmap of the odor profiles in the FMM and IMM group. (**B**): the score-radar map of the odor profiles in the FMM and IMM group. (**C**): Principal component analysis (PCA) biplot of the odor profiles in the FMM and IMM group. S1, aromatic compounds; S2, sulfides; S3, hydrogen and combustible gases; S4, broad-spectrum polar volatiles: organic acid esters, terpenes, and microbial fermentation products; S5, biogenically derived compounds and Maillard reaction products; S6, sulfur-containing organic compounds; S7, oxygenated derivatives of aliphatic hydrocarbons; S8, nitrogen oxides, ammonia, and low-molecular-weight amines; S9, aliphatic hydrocarbons; S10, total volatile organic compounds; S11, alcohols and organic solvents; S12, alkenes; S13, broad-spectrum volatiles from thermal food processing. FMM: the muscle of male crab fed with formulated feed; IMM: the muscle of male crab fed with iced trash fish.

Figure 2 shows the muscle odor profiles of male *E. sinensis*. The sensors had different responses to the volatile compounds of the different crab samples. According to Figure 2A,B, higher scores were observed in the muscle of male crab fed with formulated feed (FMM) compared to the muscle of male crab fed with iced trash fish (IMM), mainly from sensors for aromatic compounds (S1), biogenically derived compounds and Maillard reaction products (S5), sulfur-containing organic compounds (S6), aliphatic hydrocarbons (S9), total volatile organic compounds (S10), alcohols and organic solvents (S11), and alkenes (S12), which suggested that these seven sensors had better capabilities of distinguishing the male *E. sinensis* fed with formulated feed and iced trash fish.

To visualize and explore the overall differences in odor profiles among the male E. sinensis samples, a PCA plot was generated (Figure 2C). The first two principal components (PC1 and PC2) explained 97.32% of the total variance. The score plot revealed a clear separation trend between the two dietary groups along PC1.

### 3.3. Differences in Volatile Compounds in the Muscle of the Crab

**Figure 3:**
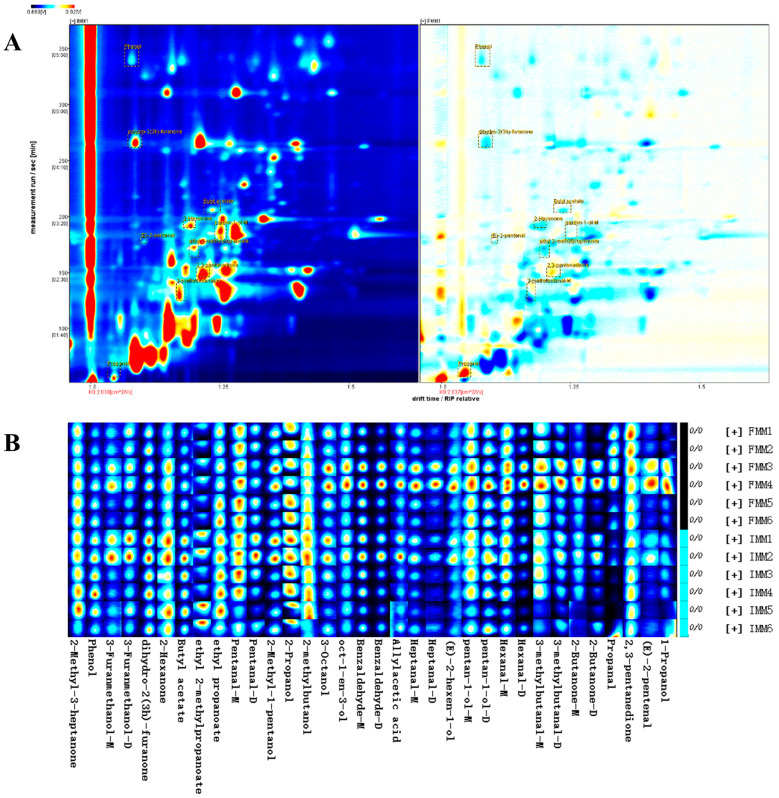
The volatiles in the crab muscle were detected by GC−IMS. (**A**): two-dimensional topographic images of the volatile substances in the muscle. IMM was selected as the reference, red spots in the FMM group meant the higher concentration to the reference, while blue spots meant the lower concentration to the reference. (**B**): the fingerprint chromatogram of the volatile substance in the muscle, the redder the color meant the higher the concentration of the volatile substance. FMM: the muscle of male crab fed with formulated feed; IMM: the muscle of male crab fed with iced trash fish.

Muscle samples from the crab fed with FF and IF were analyzed by GC–IMS. The GC–IMS Library identified a total of 58 typical target compounds from topographic plots and 34 typical target compounds (Figure 3 and Table 2). Table 2 shows the basic information of the determined compounds, which are essential for establishing an *E. sinensis* database. Twelve aldehydes, five ketones, eleven alcohols, four esters, and two acids were identified in the muscle samples, and the FMM group showed significantly higher 3-methylbutanal-M, propanal, (E)-2-pentenal, 2,3-pentanedione, and Pentan-1-ol-M, but lower 2-hexanone, dihydro-2(3h)-furanone, butyl acetate, ethyl 2-methylpropanoate, and phenol compared to IMM.

In accordance with Figure 3A, the ion migration time and the reactive ion peak (RIP) location were standardized, and the whole spectrum reflected the total headspace chemicals of the samples. RIP was represented by the red line parallel to the y-axis with an x-axis scale of 1.0. Each dot on the right side of RIP represented a volatile chemical isolated from the samples. The retention duration ranged from 100 to 350 s, whereas the drift time ranged from 1.0 to 1.5 s. The topography plot of IMM was chosen as a reference, and the gap in the topographic plot of the FMM samples was emphasized by comparing it to the reference. The color indicated the concentration of matter, with blue representing the lowest concentration and red signifying the highest. The significantly different substances were also marked in Figure 3B (*p* < 0.05). Some signals gradually disappeared as the crabs were fed different diets, and many different signals appeared simultaneously. The spectral signals of muscle in the FMM group were more abundant than those in the IMM group. The two-dimensional GC–IMS spectra offered precise and comprehensive information on the properties and intensities of aroma molecules.

**Figure 4:**
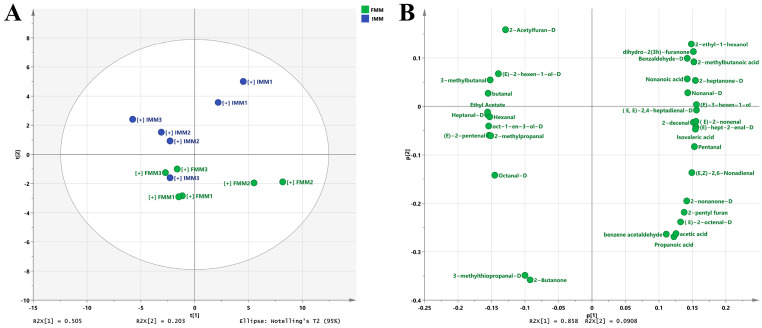
PCA biplot carried out with the volatile compounds identified in the muscle of male *Eriocheir sinensis* fed with formulated feed replacing iced trash fish. (**A**): PCA of the volatiles. (**B**): PCA loadings of the volatiles. FMM: the muscle of male crab fed with formulated feed; IMM: the muscle of male crab fed with iced trash fish.

To visualize the relationship between diet types and the muscle volatile composition, PCA was performed (Figure 4). The PCA score plot displayed distinct clustering patterns for the two groups, with minimal overlap observed between them, indicating notable differences in their overall volatile profiles.

### 3.4. Differences in Volatile Compounds in the Intestine of the Crab Fed with Formulated Feed and Iced Trash Fish

**Figure 5:**
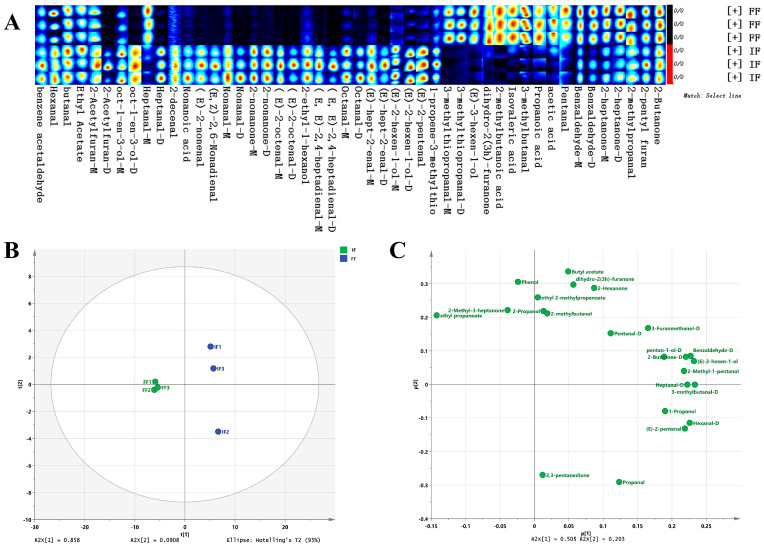
The volatiles in the intestine of the crab fed with formulated feed (FF) and iced trash fish (IF). (**A**): the fingerprint chromatogram generated using the Gallery Plot, the redder the color meant the higher the concentration of the volatile substance. (**B**): PCA of the volatiles. (**C**): PCA loadings of the volatiles.

According to Figure 5 and Table S1, a total of 48 typical target compounds from topographic plots were identified, including 27 aldehydes, 5 ketones, 6 alcohols, 2 esters, 4 acids, and 4 others. The FF group showed significantly higher levels of benzaldehyde-D, 3-methylthiopropanal-M, 3-methylthiopropanal-D, heptanal-M, 2-methylpropanal, 3-methylbutanal, pentanal, 2-heptanone-M, 2-heptanone-Da, (E)-3-hexen-1-ol, dihydro-2(3h)-furanone, 2-methylbutanoic acid, isovaleric acid, propanoic acid, and acetic acid than the IF group (*p* < 0.05). 2-butanone, ethyl acetate, 2-pentyl furan, 2-acetylfuran-M, and 2-acetylfuran-D showed no significant differences, while the contents of the remaining volatiles in IF were higher than those in FF (*p* < 0.05).

According to the PCA, the volatile compounds in crab intestines revealed distinct compositional differences between dietary groups. The R-value ranged from −1 to 1 and reflected the relative magnitude of between-group differences compared to within-group differences. The larger the absolute value of R, the more significant the between-group differences. The *p*-value referred to the significance level calculated through permutation tests. The smaller the *p*-value, the more credible the results.

### 3.5. Correlation Analysis of the Odor Profiles and Volatiles

**Figure 6:**
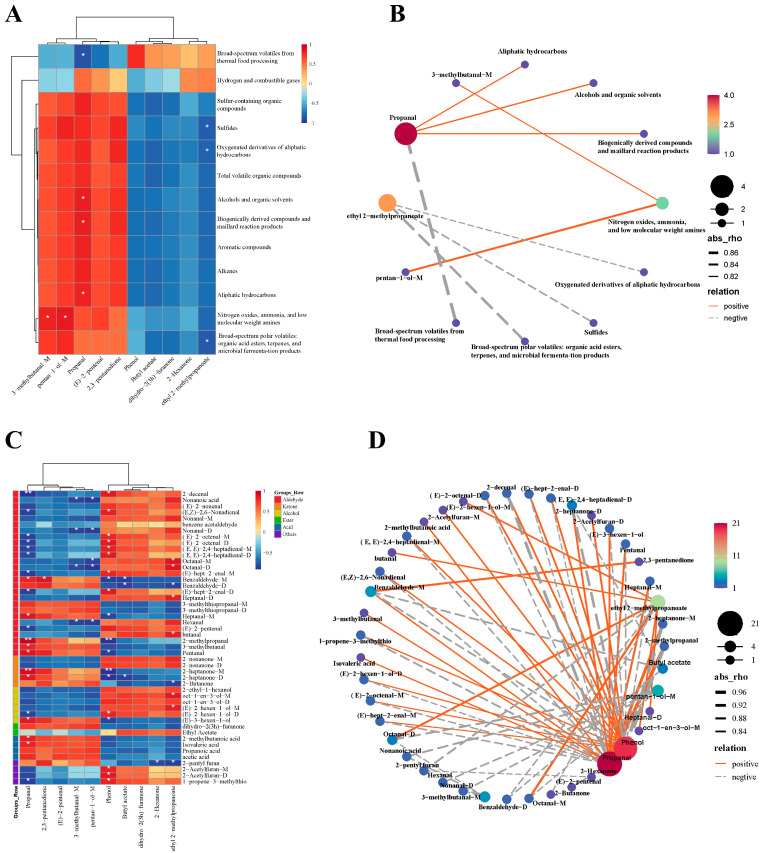
Association map of the odor profiles and volatiles of crab muscle. (**A**,**B**) are clustering correlation heatmap and correlation network between the various volatiles and the odor profiles of the muscle samples. (**C**,**D**) are clustering correlation heatmap and correlation network of the various volatiles between the muscle and diets. FMM: the muscle of male crab fed with formulated feed; IMM: the muscle of male crab fed with iced trash fish. In (**C**), * indicates a significant difference (*p* < 0.05), and ** indicates a highly significant difference (*p* < 0.01).

A Pearson correlation test was used to evaluate the correlation between the various volatiles and the odor profiles of the muscle samples. As demonstrated in Figure 6A, the cluster of 3-methylbutanal-M and pentan-1-ol-M was significantly positively correlated with nitrogen oxides, ammonia, and low-molecular-weight amines; the cluster of propanal was significantly positively correlated with alcohol, biogenically derived compounds and Maillard reaction products, and aliphatic hydrocarbons, but negatively correlated with volatile gases during food cooking; the cluster of ethyl 2-methylpropanoate was significantly negatively correlated with sulfides, oxygenated derivatives of aliphatic hydrocarbons, and broad-spectrum polar volatiles (organic acid esters, terpenes, and microbial fermentation products). Correlation networks of the various volatiles and the odor profiles of the muscle are shown in Figure 6B. The network was visualized as circles with nodes of different sizes according to the node strength. Lines joining the nodes represent positive (solid curve) and negative (dashed curve) correlations. Only relatively strong correlations (|rho| > 0.8, *p* < 0.05) were shown in the correlation networks. Propanal and ethyl 2-methylpropanoate accounted for the largest and second-largest proportions of the correlation networks. Propanal exhibited the strongest negative correlation with volatile gases during food cooking, whereas pentan-1-ol-M showed the strongest positive correlation with nitrogen oxides, ammonia, and low-molecular-weight amines.

As shown in Figure 6C, D, propanal and phenol accounted for the largest and second-largest proportions of the correlation networks, respectively. Propanal in the muscle exhibited a significant positive correlation with five aldehydes, two ketones, one alcohol, and two acids, but a significant negative correlation with nine aldehydes, one alcohol, and one other in the chyme. Phenol in the muscle exhibited a significant positive correlation with eight aldehydes, one alcohol, and three others, but a significant negative correlation with four aldehydes, two ketones, and one alcohol in the chyme.

## 4. Discussion

The sensory evaluation results in this study indicated that feed type significantly influenced the flavor profile of Chinese mitten crab muscle. Crabs in the formulated feed group (FMM) exhibited a more pronounced sweet taste, whereas those in the iced trash fish group (IMM) scored higher in saltiness and meaty flavor. Umami and sweetness are currently widely favored by consumers. The observed enhancement of sweet taste in the formulated feed group indicates that its flavor profile aligns with current consumer preferences. In contrast, the increased saltiness and meaty flavor in the iced trash fish group may collectively contribute to the complex flavor profile of the crab. However, further investigation is needed to assess its market reception [21]. First, the enhanced sweetness in the FMM group might be closely associated with the high response values of the aromatic compound (S1) and biogenically derived compounds and Maillard reaction products (S5) sensors in the electronic nose detection. Specifically, aromatic compounds such as aldehydes (e.g., benzaldehyde, octanal) and esters (e.g., ethyl acetate) often exhibit sweet or fruity aroma characteristics, which can significantly enhance the overall flavor perception of meat [22]. Furthermore, the Maillard reaction, as a key pathway for flavor formation during meat cooking, generates products such as pyrazines that possess intense sweet- and caramel-like notes, serving as important contributors to muscle sweetness [23]. It is noteworthy that the heatmap analysis in Figure 2A revealed that the IMM group showed significantly lower scores corresponding to sulfur-containing organic compounds (S6 sensor) compared to the FMM group. This difference may explain the more intense meaty flavor in the IMM group. While appropriate levels of sulfur-containing organic compounds generally enhance meaty flavor positively, excessive amounts may mask other flavor substances, leading to an overpowering or unbalanced meat taste [24]. Therefore, future research should focus on establishing an optimal range for sulfur-containing organic compounds. This could be achieved through dose–response studies that combine precise analytical chemistry (e.g., GC-MS) with trained sensory panels to model the relationship between compound concentration and sensory attributes like meaty flavor, sweetness, and umami, thereby identifying the concentration window that maximizes overall flavor acceptability.

According to previous studies, the E-nose could separate the different edible parts of the Chinese mitten crab clearly and easily [14,15]. Hence, the E-nose was used in the present study to distinguish the muscle flavor of *E. sinensis* fed with different diets. The present study showed that sensors for aromatic compounds, biogenically derived compounds and Maillard reaction products, sulfur-containing organic compounds, hydrocarbons, total volatile organic compounds, alcohols and organic solvents, and alkenes had better capabilities for distinguishing the male *E. sinensis* fed with formulated feed and iced trash fish. A similar result was also found in a previous study by sensory evaluation, which showed that the muscle of *E. sinensis* fed with FF showed higher scores of sweet taste, umami taste, and overall taste than that fed with trash fish and freshwater snails [7]. The PCA plot also revealed a distinct separation in odor profiles between crabs fed with formulated feed and those fed with iced trash fish, supporting the findings from the E-nose sensors. The main flavor substances in the muscle of the *E. sinensis* include amino acids (e.g., glutamic acid and glycine for umami and sweetness, aspartic acid, alanine), nucleotide degradation products (e.g., inosine 5′-monophosphate (IMP), which synergizes with glutamic acid to enhance umami), and volatile compounds (e.g., aldehydes such as hexanal and nonanal (grassy, fatty notes), ketones like 2,3-butanedione (buttery), and sulfur-containing compounds such as dimethyl sulfide (seafood-like)), in which volatile compounds are the main factors determining the aroma characteristics of *E. sinensis* [14,25]

The present study indicated that the muscle of *E. sinensis* fed with formulated feed and iced trash fish had different volatile flavors, and aldehydes and alcohols were the primary flavor substances in muscle samples. According to previous studies, different feeding modes (formulated feed, natural feed, trash fish) affected the content of volatile and non-volatile compounds in the edible tissues of Chinese mitten crabs, thus affecting their flavor [3,7]. Compared to compound feed, feeding fresh trash fish changed the amino acid metabolism and intestinal microbial community of Chinese mitten crabs [26].

Aldehyde compounds were the largest group of volatiles, with 12 compounds in the crab flesh samples. Linear aldehydes in the muscle are mainly generated by thermal degradation of unsaturated fatty acids and alkene peroxides. In contrast, branched aldehydes are mainly generated by oxidative deamination and decarboxylation of amino acids, and then by the Strecker degradation pathway [17]. In the present study, three saturated aldehydes (3-methylbutanal-M, propanal, and (E)-2-pentenal) in the FMM group were significantly higher than those in the IMM group, which might contribute to the aromatic taste and the flavor characteristics of sulfur-containing organic compounds. Related studies also believed that aldehydes have lower olfactory thresholds and unique odor characteristics and contribute the most to the flavor of meat [27,28]. Similar results in chicken confirmed that 3-methylbutanal is a crucial aroma substance in meat and contributes to the primary meat flavor [29]. Propanal was also found in caramel-, cooked-, and brothy-flavored chicken, which might be responsible for aroma and umami [30]. However, (E)-2-pentenal was proposed as a potential, indirect biomarker of bitter flavor [31]. The elevated levels of these aldehydes in the FMM group might stem from the metabolic oxidation of unsaturated fatty acids derived from the formulated feed, as various unsaturated fatty acids are prone to oxidative reactions yielding aldehydes such as propanal [32]. These aldehydes, with distinct aromatic properties (e.g., grassy, fruity, or caramel-like), often enhance the perception of sweetness. These findings also corroborate the results obtained from the sensory evaluation in this study [33].

In mitten crab muscle samples, alcohols were the second-most volatile substances after aldehydes. Straight-chain fatty alcohols and most branched-chain alcohols are mainly produced by lipid oxidation and microbial degradation of branched-chain aldehydes [34]. Alcohols are generally considered to be important contributors of the fatty taste and they are also closely related to the aroma of meat. Therefore, alcohol is an important part of meat flavor [35]. In the present study, only the content of pentan-1-ol-M was significantly increased by the formulated feed, which is produced by the breakdown of linoleic acid [36]. This substance is one of the main flavor components of Greek feta cheese [37] and is called a warmed-over flavor compound [38].

A total of six ketones were detected in the present study. These compounds are often associated with pleasant flavor profiles such as creamy, dairy, and fruity flavors [17]. The significant values were found in 2-hexanone and 2,3-pentanedione contents. IFM showed higher 2-hexanone, while FFM showed higher 2,3-pentanedione content. Zhang et al. proved that the aroma threshold of ketone compounds is generally high and the contributions of 2-hexanone and 2,3-pentanedione in flavor remain to be further studied [39]. Ketones, similar to aldehydes, often possess distinct aromatic properties that can enhance the perception of sweetness in the FMM group [33].

Dihydro-2(3h)-furanone, butyl acetate, and ethyl 2-methylpropanoate were the three esters detected in crab muscle. Esters are mainly produced in vivo by the esterification of carboxylic acids and alcohols produced by lipid metabolism. Esters are generally fruity and sweet. According to previous studies, pleasant “green”, “sour”, and “fruity” aromas from butyl acetate were detected in gonads of shellfish [40]. Ethyl 2-methylpropanoate is one of the primary sweet, floral, and fruity aromas in muskmelon [41]. Dihydro-2(3h)-furanone is highly correlated with sweet and fruity taste [42]. Two kinds of acids were detected in this study. The content of phenol in the IFM group was higher than that in the FFM group. Phenols in foods generally contribute to smoke-related flavor [43], but their effect in crab muscle also remain to be further researched. The increased levels of dihydro-2(3h)-furanone, butyl acetate, and ethyl 2-methylpropanoate in the IMM group is generally associated with lipid oxidation and amino acid degradation [44]. Specifically, dihydro-2(3h)-furanone contributes caramel and creamy notes [45], while esters like butyl acetate and ethyl 2-methylpropanoate provide fruity and floral aromas [46]. The richer lipid and protein content in trash fish likely facilitated the generation of these flavor compounds, collectively enhancing the meaty flavor.

According to studies on ruminant and pork, meat volatiles can be directly transferred from the diet ingested by animals into tissues, a route derived from the transformation of feed molecules by the action of gastrointestinal microorganisms. For example, intestinal microbiota can degrade complex carbohydrates in feed, such as cellulose and hemicellulose, to produce short-chain fatty acids (SCFAs). These SCFAs are absorbed into the bloodstream and subsequently deposited in tissues [11,47]. In *E. sinensis*, intestinal microorganisms could affect the fresh and sweet taste of mitten crab meat by producing amino acids, nucleotides, and other substances. Fermented feed can increase the fresh and sweet taste of mitten crab meat by changing the intestinal microbial community [48,49]. Gao et al. confirmed the important role of effective microorganisms in the overall flavor quality of edible tissues in female crabs [50]. The flavor of mitten crabs is the result of the interaction between their complex microbial community and the host’s metabolic processes. In-depth research on the dynamic changes in the mitten crab microbiome under different feeding conditions can help optimize their farming management and improve product quality [51]. To further explore the deposition law of flavor substances in *E. sinensis*, the volatiles in the intestines of the crab fed with formulated feed and iced trash fish were detected by GS-IMS. According to Zou et al., IF has higher levels of eicosapentaenoic acid and docosahexaenoic acid than FF but lower levels of oleic acid, linoleic acid, and palmitic acid [2]. Intestinal microbes also participate in producing flavor precursors or the metabolism and degradation of aromatic compounds [52]. This might explain why the unsaturated aldehydes and alcohols in IF were higher than those in FF. The volatile substances with higher content in FF are mostly saturated aldehydes, ketones, alcohols, acids, and esters.

According to the Pearson correlation test, propanal and ethyl 2-methylpropanoate accounted for the largest and second-largest proportions of the correlation networks. Propanal exhibited the strongest negative correlation with volatile gases during food cooking, while pentan-1-ol-M showed the strongest positive correlation with nitrogen oxides, ammonia, and low-molecular-weight amines. In the present study, propanal and phenol accounted for the largest and second-largest proportions of the correlation networks, respectively. The negative correlation between propanal and most unsaturated aldehydes in the intestine might be attributed to the action of intestinal microorganisms. It is further supported by the fact that propanal arises from n−3 polyunsaturated fatty acid PUFA oxidation, which is often used as a lipid oxidation indicator in foods [53]. Phenol is transformed from polyphenols in the diet by intestinal microorganisms [54]. The positive correlation between phenol and the unsaturated aldehydes might indicate that the unsaturated aldehydes were the precursors of phenolic compounds. In addition, the cluster of ethyl 2-methylpropanoate, pentan-1-ol-M, 3-methylbutanal-M, butyl acetate, 2,3-pentanedione, and 2-hexanone were significantly correlated with nine, five, four, three, one, and one various volatiles in the diets, respectively. The cluster of (E)-2-pentenal and dihydro-2(3h)-furanone had no significant correlation with the various volatiles in the diets.

The economic comparison between the two feeding strategies reveals a substantial advantage for formulated feed. The unit price of iced trash fish (IF) is approximately CNY6 per kilogram, but it exhibits a high feed conversion ratio (FCR) of about 7. In contrast, although the formulated feed (FF) is priced higher at 10 CNY/kg, it benefits from a significantly more efficient FCR of 2.5. Consequently, the production cost per kilogram of crab reaches CNY 42 when using IF, whereas it is only CNY 25 for FF. This compelling cost–benefit analysis clearly demonstrates that adopting formulated feed substantially reduces the farming cost of Chinese mitten crab compared to relying on iced trash fish.

While this study revealed compelling correlations between diet, intestinal volatiles, and muscle flavor, the underlying mechanisms require further validation. Targeted follow-up studies are needed to test the hypotheses generated here. For instance, GC-Olfactometry (GC-O) and odor-activity value (OAV) calculations could identify the key aroma-active compounds. Short-chain fatty acid (SCFA) profiling and 16S/shotgun metagenomic sequencing could directly link specific microbial taxa and their metabolic functions to the synthesis of critical flavor compounds, moving from correlation toward causation.

## 5. Conclusions

The present study investigated the muscle flavor of male *E. sinensis* fed with formulated feed replacing iced trash fish by E-nose and GC–IMS, and revealed the correlation of volatiles between the intestinal chyme and muscle. The results indicated that the crabs fed with formulated feed showed higher scores in sensors for aromatic compounds, broad-spectrum polar volatiles (e.g., terpenes), sulfur-containing organic compounds, aliphatic hydrocarbons, total volatile organic compounds, alcohols and organic solvents, and alkenes. GC–IMS identified five elevated volatiles in FF muscle (3-methylbutanal-M, propanal, (E)-2-pentenal, 2,3-pentanedione, pentan-1-ol-M) and five reduced volatiles (2-hexanone, dihydro-2(3H)-furanone, butyl acetate, ethyl 2-methylpropanoate, phenol). Propanal and ethyl 2-methylpropanoate were the dominant odor contributors based on correlation network analysis. Significant volatile correlations existed between intestinal chyme and muscle, indicating direct or microbially mediated flavor transfer. The results indicated that using a formular diet with fishmeal and soybean meal as the primary protein sources, combined with fish oil and soybean oil as the main lipid sources, as a replacement for iced trash fish during the fattening phase of *E*. *sinensis* cultivation, provides substantial benefits. These advantages encompass not only enhanced product flavor profiles, but also greater economic efficiency. Based on the comprehensive findings of this study, which demonstrated that formulated feed offers superior environmental sustainability and cost-effectiveness compared to iced trash fish while actively enhancing crab flavor quality, we advocate for its adoption in Chinese mitten crab aquaculture practices.

## Figures and Tables

**Table 1:** The ingredients and proximate composition of experimental diets (%, air-dry basis).

Ingredients		Proximate Composition	
Fishmeal ^1^	32.00	Crude protein	39.72
Soybean meal ^1^	10.00	Crude lipid	7.69
Rapeseed meal ^1^	10.00	Ash	10.02
Peanut meal ^1^	10.00	Gross energy (MJ/kg)	18.49
Blood meal ^1^	6.00		
α-starch ^2^	22.50		
Fish oil ^1^	2.00		
Soybean oil ^1^	2.00		
Monocalcium phosphate ^1^	2.00		
Choline chloride (50%) ^3^	1.00		
Vitamin and mineral premix ^3^	1.00		
Bentonite ^3^	1.00		
Salt	0.50		

Notes: ^1^ Obtained from Jiangsu Fuyuda Food Products Co., Ltd., Yangzhou, China; ^2^ Obtained from Foshan Guonong Starch Co., Ltd., Foshan, China; ^3^ Obtained from Wuxi Hanove Animal Health Products Co., Ltd., Wuxi, China.

**Table 2:** GC–IMS integration parameters of volatile compounds in the muscle of *Eriocheir sinensis*.

Volatiles	NO.	Compounds	CAS#	Formula	RI	Retention Time (s)	Drift Time (ms)	Intensity (Volume)	
								FMM	IMM
Aldehydes	1	Benzaldehyde-M	C100527	C_7_H_6_O	957.7	311.3	1.1	588.8 ± 54.5	544.5 ± 44.7
	2	Benzaldehyde-D	C100527	C_7_H_6_O	957.0	310.8	1.5	213.0 ± 41.9	202.0 ± 30.9
	3	Heptanal-D	C111717	C_7_H_14_O	899.3	260.8	1.7	74.2 ± 15.5	62.2 ± 7.8
	4	Hexanal-D	C66251	C_6_H_12_O	791.5	198.1	1.6	497.3 ± 112.3	284.0 ± 51.5
	5	Hexanal-M	C66251	C_6_H_12_O	791.8	198.3	1.3	565.4 ± 33.4	454.6 ± 48.3
	6	Pentanal-M	C110623	C_5_H_10_O	698.6	152.8	1.2	435.2 ± 17.3	364 ± 46.1
	7	Pentanal-D	C110623	C_5_H_10_O	700.2	153.5	1.4	492.9 ± 19.5	547 ± 90.5
	8	3-methylbutanal-M	C590863	C_5_H_10_O	654.1	132.5	1.2	723.0 ± 42.1 ^a^	501.8 ± 79.7 ^b^
	9	3-methylbutanal-D	C590863	C_5_H_10_O	656.9	133.8	1.4	1628.7 ± 292.8	1386.4 ± 188.2
	10	Heptanal-M	C111717	C_7_H_14_O	900.4	261.7	1.3	245.2 ± 36	182.1 ± 19
	11	Propanal	C123386	C_3_H_6_O	493.4	60.9	1.0	809 ± 108.3 ^a^	308.3 ± 112.8 ^b^
	12	(E)-2-pentenal	C1576870	C_5_H_8_O	752.4	178.7	1.1	38.7 ± 7.1 ^a^	19.8 ± 3.7 ^b^
Ketones	1	2-Methyl-3-heptanone	C13019200	C_8_H_16_O	1094.6	895.9	1.3	636.1 ± 17.0	688.0 ± 53.0
	2	2-Hexanone	C591786	C_6_H_12_O	781.6	192.8	1.2	211 ± 17.7 ^b^	268.2 ± 17.6 ^a^
	3	2-Butanone-M	C78933	C_4_H_8_O	598.7	107.9	1.1	547.8 ± 76.7	406.1 ± 75.2
	4	2-Butanone-D	C78933	C_4_H_8_O	597.8	107.4	1.2	1163.2 ± 423.1	1234.3 ± 266.1
	5	2,3-pentanedione	C600146	C_5_H_8_O_2_	693.4	150.3	1.2	726.3 ± 40.5 ^a^	615.6 ± 18.1 ^b^
Alcohols	1	3-Octanol	C589980	C_8_H_18_O	998.6	356.6	1.4	253.7 ± 22.7	225.3 ± 21.4
	2	Oct-1-en-3-ol	C3391864	C_8_H_16_O	982.0	332.4	1.2	403.9 ± 35.1	331.0 ± 23.4
	3	3-Furanmethanol-M	C4412913	C_5_H_6_O_2_	973.7	325.2	1.1	119.6 ± 15.8	130.0 ± 18.2
	4	3-Furanmethanol-D	C4412913	C_5_H_6_O_2_	974.2	325.7	1.4	213.1 ± 18.8	226.7 ± 28.2
	5	(E)-2-hexen-1-ol	C928950	C_6_H_12_O	849.3	230.6	1.2	172.7 ± 19.8	155.5 ± 18.0
	6	Pentan-1-ol-D	C71410	C_5_H_12_O	764.0	184.3	1.5	474.1 ± 31.6	477 ± 50.5
	7	Pentan-1-ol-M	C71410	C_5_H_12_O	767.1	185.8	1.3	732.7 ± 26.2 ^a^	573.7 ± 51.2 ^b^
	8	2-Methyl-1-pentanol	C105306	C_6_H_14_O	847.0	229.2	1.3	325.6 ± 33.7	290.6 ± 41.2
	9	2-Propanol	C67630	C_3_H_8_O	524.8	74.9	1.1	4227.3 ± 293.9	4647 ± 541
	10	2-methylbutanol	C137326	C_5_H_12_O	731.5	168.6	1.2	152.4 ± 6.3	155.4 ± 14.4
	11	1-Propanol	C71238	C_3_H_8_O	571.0	95.5	1.1	287.6 ± 70.1	245.2 ± 31.3
Esters	1	Dihydro-2(3h)-furanone	C96480	C_4_H_6_O_2_	904.8	265.5	1.1	465.7 ± 24.2 ^b^	598.6 ± 38.0 ^a^
	2	Butyl acetate	C123864	C_6_H_12_O_2_	805.8	206.2	1.2	190.6 ± 5.6 ^b^	241.8 ± 17.5 ^a^
	3	Ethyl 2-methylpropanoate	C97621	C_6_H_12_O_2_	733.5	169.6	1.2	200 ± 10.9 ^b^	308.6 ± 27.0 ^a^
	4	Ethyl propanoate	C105373	C_5_H_10_O_2_	713.7	160.1	1.2	790.0 ± 50.3	856.3 ± 46.3
Acids	1	Allylacetic acid	C591800	C_5_H_8_O_2_	897.4	259.1	1.1	208.5 ± 12.0	202.4 ± 12.9
	2	Phenol	C108952	C_6_H_6_O	991.6	340.7	1.1	245.6 ± 11.3 ^b^	317.5 ± 19.3 ^a^

Notes: FMM, the muscle of the male crab fed with formulated feed (n = 3 ponds); IMM, the muscle of the male crab fed with iced trash fish (n = 3 ponds). Different letters among the different groups mean the significant difference (*p* < 0.05).

## Data Availability

The original contributions presented in the study are included in the article, further inquiries can be directed to the corresponding authors.

## Supplementary Materials

The following supporting information can be downloaded at: https://www.mdpi.com/article/10.3390/ani15213101/s1, Table S1: GC-IMS integration parameters of volatile compounds in the intestine of *Eriocheir sinensis* fed with formula feed (FF) and ice fish (IF).
