# Supplementary material for: Enhanced Muscle Flavor in Male Chinese Mitten Crab (Eriocheir sinensis) Driven by Feed-Induced Reconfiguration of Intestinal Volatile Compounds
Source: Animals (Basel). 2025 Oct 25;15(21):3101. doi: 10.3390/ani15213101 (PMC12609694; doi:10.3390/ani15213101)
Supplement: Supplementary file 1 [file animals-15-03101-s001.zip › animals-3891227-supplementary.pdf]

Table S1 GC-IMS integration parameters of volatile compounds in the intestine of *Eriocheir sinensis* fed with formula feed (FF) and ice fish (IF)

Volatiles	NO.	Compounds	CAS#	Formula	RI	Retention time (s)	Drift time (ms)	Intensity (volume)		*P* Value
								FF	IF	
Aldehydes	1	2-decenal	C3913711	$C_{10}H_{18}O$	1408.80	928.72	1.48	155.3±18.4	297.5±11.0	0.003
	2	Nonanoic acid	C112050	$C_9H_{18}O_2$	1278.60	743.42	1.55	154.9±11.0	508.3±75.7	0.040
	3	(E)-2-nonenal	C18829566	$C_9H_{16}O$	1192.40	620.84	1.41	98.6±11.9	754.3±52.3	0.000
	4	(E,Z)-2,6-Nonadienal	C557482	$C_9H_{14}O$	1178.70	601.37	1.37	100.5±6.6	926.9±160.0	0.035
	5	Nonanal-M	C124196	$C_9H_{18}O$	1111.80	506.20	1.49	313.2±13.3	1585.8±116.0	0.008
	6	Benzene acetaldehyde	C122781	C_8H_8O	1041.20	405.78	1.26	349.2±6.5	415.9±36.9	0.149
	7	Nonanal-D	C124196	$C_9H_{18}O$	1111.00	505.10	1.95	67.6±2.9	939.3±195.2	0.047
	8	(E)-2-octenal-M	C2548870	$C_8H_{14}O$	1056.10	426.94	1.33	140.7±9.6	1707.5±159.0	0.010
	9	(E)-2-octenal-D	C2548870	$C_8H_{14}O$	1056.10	426.94	1.82	52.4±2.6	684.5±176.9	0.070
	10	(E, E)-2,4-heptadienal-M	C4313035	$C_7H_{10}O$	1015.40	369.07	1.20	276.5±3.3	2248.5±258.3	0.017
	11	(E, E)-2,4-heptadienal-D	C4313035	$C_7H_{10}O$	1013.30	366.05	1.62	106.8±9.1	2596.5±898.7	0.109
	12	Octanal-M	C124130	$C_8H_{16}O$	1006.30	356.15	1.42	309.9±7.6	1266.6±122.2	0.001
	13	Octanal-D	C124130	$C_8H_{16}O$	1006.20	355.95	1.82	130.2±46.4	1715.4±266.1	0.004
	14	(E)-hept-2-enal-M	C18829555	$C_7H_{12}O$	958.10	310.29	1.25	68.3±8.0	343.3±13.4	0.000
	15	Benzaldehyde-M	C100527	C_7H_6O	960.20	311.96	1.15	838.3±52.4	517.5±60.7	0.016
	16	Benzaldehyde-D	C100527	C_7H_6O	959.80	311.68	1.47	756.1±29.1	307.8±63.9	0.003
	17	(E)-hept-2-enal-D	C18829555	$C_7H_{12}O$	955.50	308.06	1.67	54.7±2.8	680.3±198.2	0.087
	18	Heptanal-D	C111717	$C_7H_{14}O$	901.50	263.16	1.70	149.7±13.2	1697.0±135.4	0.000
	19	3-methylthiopropanal-M	C3268493	C_4H_8OS	907.00	267.74	1.09	485.7±20.5	36.6±4.1	0.001
	20	3-methylthiopropanal-D	C3268493	C_4H_8OS	905.70	266.66	1.40	251.1±18.6	59.0±7.1	0.001
	21	Heptanal-M	C111717	$C_7H_{14}O$	899.50	261.48	1.33	170.7±15.5	50.9±8.5	0.002
	22	Hexanal	C66251	$C_6H_{12}O$	794.60	202.74	1.56	876.8±106.1	1596.7±101.3	0.008
	23	(E)-2-pentenal	C1576870	C_5H_8O	752.90	184.71	1.36	1366.6±38.7	5894±391.9	0.007

	24	Butanal	C123728	C_4H_8O	600.60	133.95	1.29	1682.9±49.3	1978.0±41.3	0.010
	25	2-methylpropanal	C78842	C_4H_8O	565.90	123.99	1.28	906.4±29.4	778.9±38.9	0.059
	26	3-methylbutanal	C590863	$C_5H_{10}O$	654.40	149.38	1.41	5580.0±93.2	1115.7±98.4	0.000
	27	Pentanal	C110623	$C_5H_{10}O$	695.90	162.08	1.42	251.2±22.9	107.9±5.0	0.004
Ketones	1	2-nonanone-M	C821556	$C_9H_{18}O$	1095.20	482.65	1.41	195.6±17.0	1611.0±32.9	0.000
	2	2-nonanone-D	C821556	$C_9H_{18}O$	1095.20	482.65	1.88	65.2±3.2	659.4±17.4	0.000
	3	2-heptanone-M	C110430	$C_7H_{14}O$	889.70	254.08	1.26	432.8±32.6	279.3±25.0	0.020
	4	2-heptanone-D	C110430	$C_7H_{14}O$	890.00	254.25	1.63	813.2±15.1	516.9±42.3	0.003
	5	2-Butanone	C78933	C_4H_8O	587.00	130.05	1.25	4618.9±47.5	4242.2±189.7	0.126
Alcohols	1	2-ethyl-1-hexanol	C104767	$C_8H_{18}O$	1029.10	388.50	1.27	83.7±8.6	279.1±17.4	0.001
	2	Oct-1-en-3-ol-M	C3391864	$C_8H_{16}O$	984.60	332.32	1.16	580.9±5.1	1095.4±43.1	0.006
	3	Oct-1-en-3-ol-D	C3391864	$C_8H_{16}O$	982.20	330.32	1.59	37.3±2.4	447.4±13.9	0.000
	4	(E)-2-hexen-1-ol-M	C928950	$C_6H_{12}O$	851.40	233.41	1.18	259.6±3.9	916.7±50.9	0.000
	5	(E)-2-hexen-1-ol-D	C928950	$C_6H_{12}O$	850.20	232.76	1.51	1721.3±41.2	8967.6±661.1	0.008
	6	(E)-3-hexen-1-ol	C928972	$C_6H_{12}O$	857.30	236.61	1.25	471.0±36.3	108.4±8.2	0.001
Esters	1	Dihydro-2(3h)-furanone	C96480	$C_4H_6O_2$	919.70	278.32	1.08	539.5±27.8	93.6±17.0	0.000
	2	Ethyl Acetate	C141786	$C_4H_8O_2$	613.20	137.56	1.34	515.9±23.7	572.6±63.0	0.447
Acids	1	2-methylbutanoic acid	C116530	$C_5H_{10}O_2$	845.80	230.39	1.20	213.8±7.9	23.7±5.9	0.000
	2	Isovaleric acid	C503742	$C_5H_{10}O_2$	831.90	222.87	1.22	342.8±9.6	68.6±23.7	0.000
	3	Propanoic acid	C79094	$C_3H_6O_2$	708.30	167.03	1.27	355.5±19.0	184.8±2.7	0.011
	4	Acetic acid	C64197	$C_2H_4O_2$	622.60	140.26	1.05	764.2±30.7	252.0±67.2	0.002
Others	1	2-pentyl furan	C3777693	$C_9H_{14}O$	995.90	341.73	1.25	261.0±6.8	218.7±23.8	0.162
	2	2-Acetylfuran-M	C1192627	$C_6H_6O_2$	913.80	273.39	1.11	352.0±6.4	485.8±59.9	0.154
	3	2-Acetylfuran-D	C1192627	$C_6H_6O_2$	910.10	270.36	1.45	144.3±1.5	1315.9±499.3	0.144
	4	1-propene-3-methylthio	C10152768	C_4H_8S	700.40	163.89	1.04	466.5±19.0	976.3±67.4	0.002
